# Topological characterization of neuronal arbor morphology via sequence representation: I - motif analysis

**DOI:** 10.1186/s12859-015-0604-2

**Published:** 2015-07-10

**Authors:** Todd A Gillette, Giorgio A Ascoli

**Affiliations:** Department of Molecular Neuroscience, Center for Neural Informatics, Structures, and Plasticity, Krasnow Institute for Advanced Study (MS2A1), George Mason University, Fairfax, VA USA

**Keywords:** Neuronal morphology, Tree topology, Motif analysis

## Abstract

**Background:**

The morphology of neurons offers many insights into developmental processes and signal processing. Numerous reports have focused on metrics at the level of individual branches or whole arbors; however, no studies have attempted to quantify repeated morphological patterns within neuronal trees. We introduce a novel sequential encoding of neurite branching suitable to explore topological patterns.

**Results:**

Using all possible branching topologies for comparison we show that the relative abundance of short patterns of up to three bifurcations, together with overall tree size, effectively capture the local branching patterns of neurons. Dendrites and axons display broadly similar topological motifs (over-represented patterns) and anti-motifs (under-represented patterns), differing most in their proportions of bifurcations with one terminal branch and in select sub-sequences of three bifurcations. In addition, pyramidal apical dendrites reveal a distinct motif profile.

**Conclusions:**

The quantitative characterization of topological motifs in neuronal arbors provides a thorough description of local features and detailed boundaries for growth mechanisms and hypothesized computational functions.

**Electronic supplementary material:**

The online version of this article (doi:10.1186/s12859-015-0604-2) contains supplementary material, which is available to authorized users.

## Background

Neuronal morphology is determined by a number of factors, including physical and biological constraints and requirements of axonal, dendritic, and network function. Branching topology is a complex feature of arbor morphology and is generally measured via one of several metrics: number of branches, maximum branch order (i.e. number of bifurcations between root and tip), partition asymmetry [1], and caulescence (i.e. prominence of a main path [2]). While these metrics have proven useful in many studies, they do not necessarily capture the detailed branching patterns of neurons. We introduce a method for representing a neuronal tree as a sequence of characters, each encoding for select features of a branch. We tested this method on a large set of neuronal reconstructions from NeuroMorpho.Org [3]. Specifically, we analyzed the branching sequences for motifs to identify patterns (subsequences) representative of arbor types (axons, dendrites, and pyramidal apical dendrites).

Branch diameter and neurite length have long been known to impact passive and active electrical propagation [4,5], and branching patterns can influence the order and timing of input signal integration and postsynaptic receipt [6,7]. Ultimately it is the combination of features, including non-morphological features such as channel composition and density, which determine electrophysiological function. Moreover, arbor size and branching patterns reflect the distribution of synaptic (pre or post) targets of a neuron given metabolic and volumetric constraints [8-10]. Beyond the functional focus, branching features result from particular growth processes and thus can be used to validate growth models driven by biophysics [11,12], molecular and signaling dynamics [13-16], statistical relationships [17-20], or more abstract positional and branch-type rules [21,22].

Until recently, the challenges of generating relevant biological data have limited cell type growth analyses to a single type or a few related cell types at a time. However, accelerating data production and curation of datasets from a growing number of laboratories by NeuroMorpho.Org have enabled large-scale analysis. Some studies have used these data to detect general principles of neuronal organization [10,23]. New imaging and semi- to fully-automated reconstruction processes [24,25] are contributing to increased throughput, with ever larger datasets to be expected in the near future [26]. For detecting relationships between cell types, this influx of data calls for new methods of analysis [27].

Other fields have seen similarly dramatic growth in data, most notably genomics. Statisticians and computer scientists responded by creating a wide and still growing array of techniques to sort through the data in a practical timeframe in search of significant relationships and findings. These techniques broadly include local and global alignment [28-30]; as well as multiple sequence alignment [31], each with many specific algorithms designed for targeted sensitivity and/or efficiency. We leveraged the underlying bases of these techniques to analyze neuronal morphology by representing axonal and dendritic trees as sequences of branches encoded by their features. We began by sequentially encoding local topology based on a simple binary tree representation of neuronal structure. We then applied motif analysis [32] to determine the defining topological patterns across arbor types from a broad range of species, neuron classes, and brain regions. Highly over- or under-expressed patterns constitute motifs and anti-motifs, respectively.

## Methods

All code is available open source at http://krasnow1.gmu.edu/cn3/NeuriteSequence/, including Java implementation, R analysis scripts, and related documentation.

### Neurite trees to sequences

**Figure 1:**
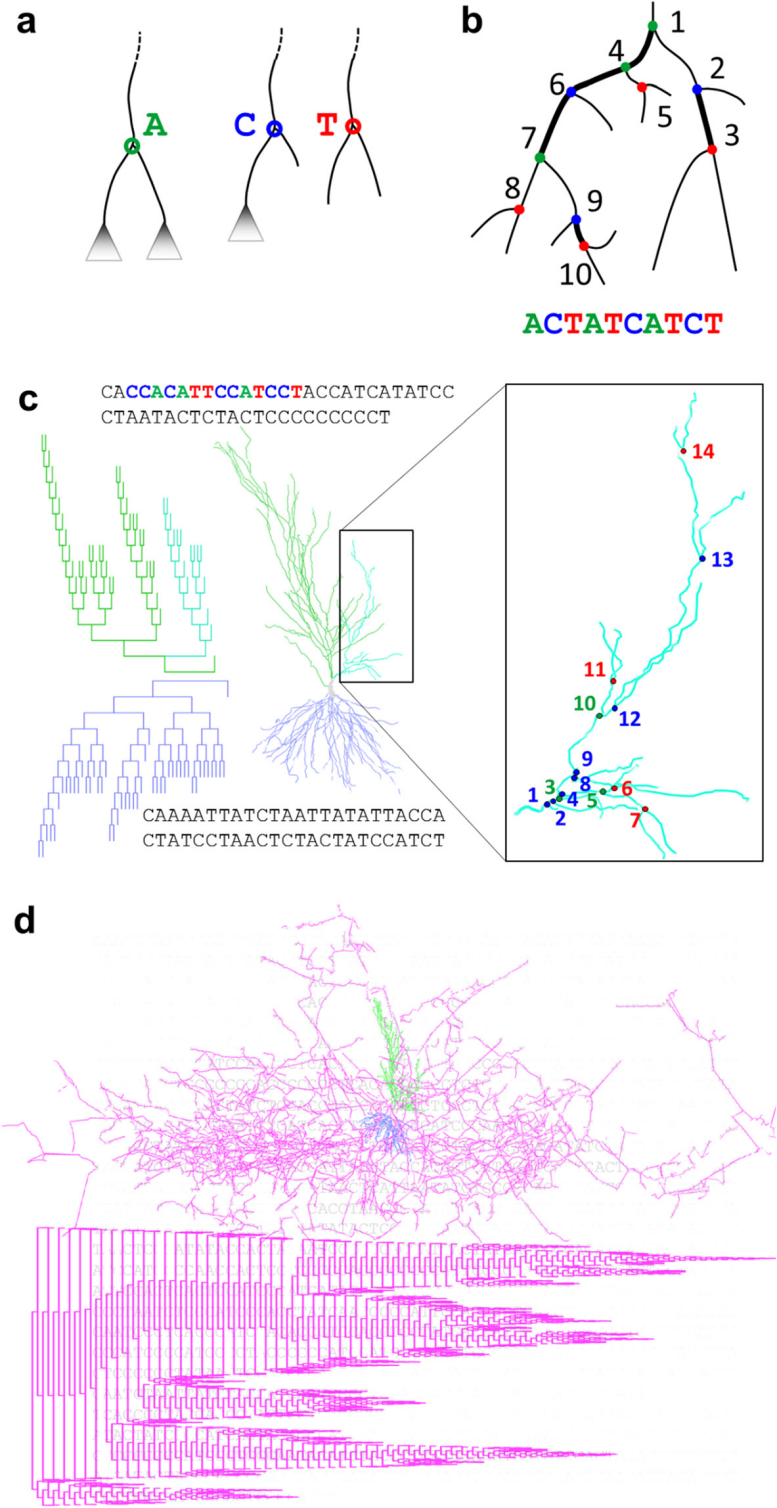
Converting tree to sequence. **a**. Bifurcation nodes are encoded as characters based on whether their child branches bifurcate or terminate. Arborization (*A*) nodes have two bifurcating children. Continuation (*C*) nodes have one bifurcating and one terminating child. Termination (*T*) nodes have two terminating children. **b**. Nodes are traversed depth-first starting from the smaller side which optimally preserves locality. **c**. Hippocampal pyramidal cell apical (green) and basal (blue) dendrograms and morphologies are shown (NMO_00191 from [62]), with enlargement of a portion of the apical dendrite (right) and coloring in the sequence. Node types are colored and numbered by their order in the sequence starting with the first node in the subtree. **d**. The entire pyramidal cell morphology is shown (top), with dendrogram (bottom) and sequence representations (background) of the axonal arbor (magenta) (NMO_07897 from [63]).

The possible ways to encode a neurite as a sequence are numerous. As the first and simplest approach, we used local topology alone for the encoding of bifurcation nodes. Specifically, bifurcations are encoded on the basis of whether their child branches lead to bifurcations or terminations. Bifurcations in which both child branches themselves bifurcate are encoded with the letter ‘*A*’ (for “arborizing”). Bifurcations with one bifurcating child and one terminating child are encoded as ‘*C*’ as the tree “continues” without adding a new subtree. Bifurcations with two terminating children are encoded as ‘*T*’ (Figure 1a). These definitions are equivalent to those used in vertex analysis [33] with the *A*, *C*, and *T* bifurcation types referred to as tertiary, secondary, and primary nodes. Note that terminal branches, though not explicitly encoded, are fully accounted for in this method.

To string the character representations into a sequence, neuronal trees must be traversed. We use a traversal that encodes the root node of a tree and then recursively selects each subtree of that root in succession (i.e. prefix, depth-first traversal). One of the child subtrees is fully traversed first, followed by the second child subtree, and the letter encoding each encountered node during traversal is appended to the sequence (Figure 1b). This method optimizes the locality of representation, keeping nodes that are adjacent in the tree structure as close as possible in the sequence. Example sequences of the apical dendrite, basal dendrite, and axon of a pyramidal cell can be seen in Figure 1c,d. The order of subtree traversal is consistently determined by the number of bifurcations in each subtree. We investigated topological sequencing produced by always traversing the smaller subtree first (Smaller then Larger: *StL*) as well as those produced by always traversing the larger subtree first (Larger then Smaller: *LtS*). The *StL* representation has a greater locality, with adjacent tree nodes being nearer in the resulting sequence, than the *LtS* representation. All sequences and *k*-mers shown here are based on *StL* traversal, though the full analysis also includes unique *LtS* *k*-mers, as discussed in Section 2.2. In either *StL* or *LtS* traversal, when both subtrees of a node have the exact same number of bifurcations, the order is determined by the subtree topology, as described by Harding [34]. Specifically, trees with higher partition asymmetry are treated as the larger subtree.

### *K*-mers

**Figure 2:**
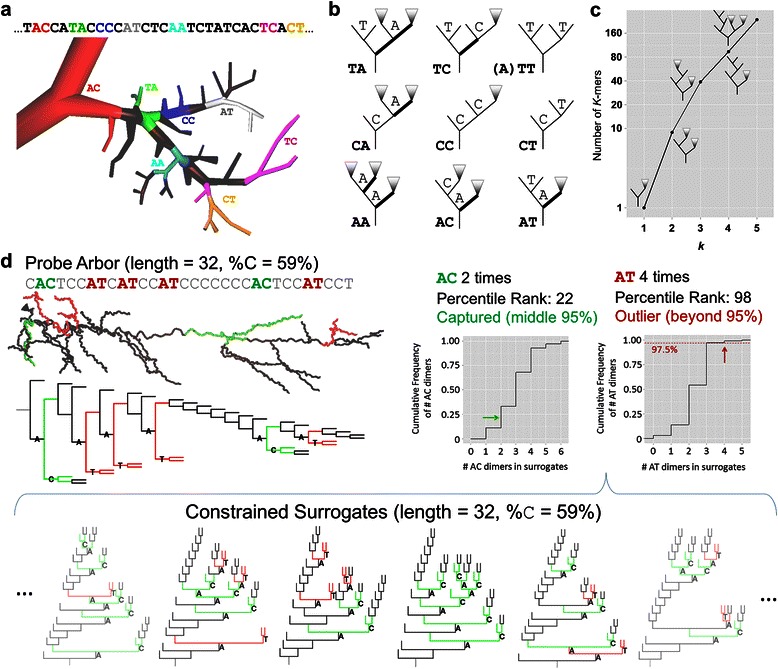
Measuring *k*-mers. **a**. Highlighted dimers in a portion of a fly tangential cell [64] and its associated sequence. **b**. Dimer schematics displaying the possible configurations. Triangles represent subtrees of unspecified size and shape. Bold segments indicate branches leading to the larger side subtree of the parent *A* node. Given the (Smaller then Larger) traversal method, the *TT* dimer must be preceded by an *A*. *TA* and *TC* schematics are examples, as additional bifurcations could be found between the parent *A* and small-side *T* nodes. **c**. Number of *k-*mers (with examples) by *k* shows an approximately exponential rise. **d**. Calculating the percentile rank of a *k*-mer given the distribution of *k*-mer counts in the source sequence’s surrogate population. An example apical dendrite (NMO_02582 from [65]), dendrogram, and sequence are shown along with cumulative distribution of *k*-mer counts for *k*-mers *AT* (red) and *AC* (green). Below: Six out of 100 node-type-constrained surrogates are shown. The example *k*-mers are highlighted and their counts compose the distributions. Colored dots show the respective percentile ranks of the apical dendrite *k-*mer counts, with *AT* being above nearly the entire surrogate distribution (thus constituting a motif) and *AC* being “captured” inside the middle 95% of its surrogate distribution.

To determine what patterns neurites exhibit among all tree shapes, a motif analysis was carried out for bifurcation subsequences of (increasing) length *k*, termed *k*-mers. Besides the three monomers *A*, *C*, and *T*, there are nine dimers (Figure 2a,b), and the number of *k*-mers grows approximately exponentially with *k* (Figure 2c). There are 27 permutations of trimer sequences, but not all exist due to tree constraints, while some *LtS* trimers are included as they capture different structures than *StL* trimers (the same applies to tetrametrs and pentamers). The *StL* trimers *CTT* and *TTT* do not occur as the latter *T* is a complete subtree that is smaller than its preceding sibling subtree. Any *LtS k*-mer with an *A* or *T* in the middle (of which there are 14 trimers), such as *AAT* or *CCTC*, describes a sequence of bifurcations not captured by any *StL k*-mer. In contrast, the *AC* *StL* dimer represents a pattern equivalent to that of the *TC* *LtS* dimer as in both cases the *C* is the smaller-side child of an *A*. The same relationship holds between the *ACT* *StL* and *TCT* *LtS* trimers, in which the *CT* is the smaller-side child of an *A*. Indeed, some *LtS* trimers do differ from their corresponding *StL* trimers (e.g. *CTC* and L-*CTC* or *ATA* and L-*TTA*: see Additional file 1: Figure S3).

Additional topological patterns involve combinations of traversals (e.g. *AAA* where the second *A* is the smaller-side child of the first, and the third is the larger-side child of the second) that are not captured in this analysis. While from the topological perspective the choice of combinations to include is arbitrary, restricting the analysis to only *LtS* and *StL* traversals is consistent with a sequence-based analysis and substantially simplifies the project design. Given the minimal pattern information beyond *k*-mers of size 3 (see section 3.2), only the mixed traversal *k*-mers *AAA* and *TAA* are left out of the analysis.

The count and the proportion (i.e. count per sequence length) of each dimer are dependent upon node type proportions. For instance, a sequence with more *C* bifurcations than another would be expected to also have more *CC* dimers. The same effect applies to measuring trimer proportions with regard to dimer proportions, and so on. In order to account for these dependences, we normalize the analysis of *k*-mers for a given neurite sequence by a set of control trees with the same distribution of (*k-1*)-mers. Specifically, a set of 100 surrogate tree sequences is generated (sampled from the entire set of tree topologies) and constrained by (*k-1*)-mer proportions and tree size (Figure 2d). This produces an expected distribution of 100 values for each sequence and *k*-mer, allowing the computation of a percentile rank (PR) for the *k*-mer proportion in the original neurite sequence. The PR provides a value comparable across *k*-mers, arbor types, and individual sequences (i.e. axons, dendrites, and pyramidal apical dendrites). A *k*-mer that occurs significantly more often across a group of neurites than in the surrogate set is considered a motif; conversely, a *k*-mer that occurs significantly less often across a group of neurites than in the surrogate set is considered an anti-motif.

Percentile rank is calculated as

$$ \frac{R-0.5}{N} $$

where *N* is the number of values in the distribution, and *R* (rank) is the position of the value in an ordered list of values in the distribution. In case of multiple instances with the given value in the distribution, the rank is given by their central index (or the mean of the central indices) of instances with the value. For example, if the first 15 values are ‘3’, then the rank of the value ‘3’ will be 8 ([1 + 15]/2), and the PR will be 7.5%. If the value is smaller than all values in the distribution, the rank will be 1 and the PR will be (1 − 0.5)/100 = 0.005 or 0.5%. If the value is larger than all values in the distribution, the rank will be 100 and the PR will be (100 − 0.5)/100 = 0.995 or 99.5%.

All statistical tests used the Wilcoxon rank sum or signed rank tests. P-values determining whether a *k*-mer was significantly different than the baseline were adjusted for multiple testing using Bonferroni correction given the number of *k*-mers.

### Constrained tree-sequence generation

An algorithm for determining the number of topologies given tree size, and generating a topology given a number within that range, was implemented based on the description in [34]. The algorithm determines the number of topologies and the specific structure of a given topology (by number) recursively. The topology number determines how the number of branches is apportioned to either subtree; the topology number of each subtree is then calculated, and each subtree undergoes the same process until any given subtree is small enough to have only one shape. An algorithm for sampling length and node-type constrained surrogates was developed based on the same principles, using the total number of bifurcations and the number of *C* bifurcations. However, no known method was found for rapid random tree generation fitting the constraints of *k*-mers with *k* ≥ 2 while ensuring that the resulting trees are uniformly sampled over the distribution of tree shapes. The enormous number of trees that need to be generated before one matching the *k*-mer constraints is found made the length and node-type-constrained algorithm impractical. Therefore, multiple programs, one each for dimer, trimer, and tetramer constrained surrogates, were written that built tree shapes with *k*-mer defined components. The resulting distributions were compared with a smaller set of those produced by accepting constraint-satisfying trees from among those generated using the algorithm for producing uniformly distributed tree shapes (specifically the node-type constrained version). Minor modifications in the code were made to minimize the small deviations in *k + 1*-mer distributions seen in the “*k*-mer built” surrogates (detailed comments provided within the code). The most extreme deviations were a median difference of 0.1% between constructed surrogate and constrained uniform surrogate proportions for trimers *CTC* and *TTC*.

### Tree growth methods

Simple growth models were used to generate trees for comparison with available data. The models are versions of the general QS-model [22]. The QS-model takes three parameters as constraints to stochastically generate a tree. A size parameter determines the number of branches. The parameter Q determines the propensity for bifurcation events to occur at terminal branches versus interstitial (non-terminal) branches. A value of 0 produces purely terminal growth, a value of 1 produces purely interstitial growth, and values of 0.5 produces segmental (uniform) growth.

The parameter S determines the branch order bias of bifurcation probability. A value of 0 means no order bias; positive and negative values produce a bias towards low- and high-order branching, respectively. Specifically, the probability of a terminal node being selected to branch at a given branch event is given by *C* × 2^− *Sγ*^, where *C* is a normalization constant and *γ* is branch order. The terminal growth model used to approximate dendrites is low-order biased, using Q = 0 and S = 0.4. The segmental growth model used has no order bias, with Q = 0.5 and S = 0.

**Figure 3:**
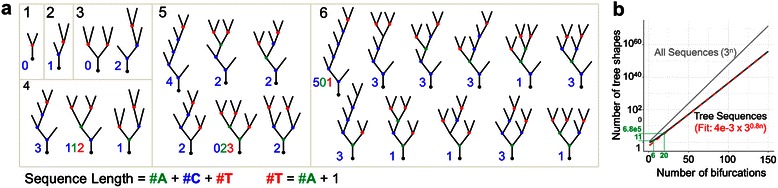
Tree size and complexity. **a**. Complexity of trees is limited by tree size. Here are shown the set of possible tree shapes for trees with 1 to 6 bifurcations. Additionally, the number of *T* nodes (red dots in sample trees) is always 1 more than *A* nodes (green dots). Thus, size and number or percent of *C* nodes (yellow dots) fully captures node-type statistics. **b**. Number of tree shapes for tree size (in # bifurcations, or sequence length). The relationship is approximately exponential, though the number is smaller than for the set of all possible sequences of the same size and alphabet unconstrained by “treeness”. Green lines indicate the 11 topologies of trees with 6 bifurcations displayed in **(a)** and the 680,000 tree shapes with 20 bifurcations which serves as the minimum complexity cutoff for the analysis.

### The NeuroMorpho.Org dataset

All data used in this study were downloaded from the NeuroMorpho.Org database (version 5.6), which houses neuronal reconstructions from a wide variety of species, brain regions, cell types, labs, and experimental methods [3]. In order to minimize confounds, we eliminated neurons cultured in non-organotypic environments (N = 29) as well as the topologically simple neurons from the OpenWorm archive (N = 302). Neurons from non-control (e.g. drug-affected) conditions were also eliminated (N = 1288), and only one of multiple reconstructions found to be traced from the same neurons were kept (N = 25 removed), bringing the dataset to 8,223 reconstructions.

Each reconstruction was partitioned into different neurite arbors (indicated by the ‘type’ column in SWC-formatted reconstruction files). Given the clear differentiation between axons and dendrites, the two arbor types were analyzed separately. Additionally, for pyramidal cells, apical dendrites were sequenced as a third distinct arbor type given its well-known morphological differences from the basal trees [20,35]. Pyramidal basal dendrites were grouped with non-pyramidal dendrites, and pyramidal neurons without differential labeling of apical and basal arbors were removed from the dataset (N = 20). Apical dendrites of non-pyramidal cells were ignored (N = 11).

Many neuron types have multiple dendritic trees, sometimes with fairly few bifurcations each. Separate dendritic trees originating from the same soma were combined into a single binary dendritic arbor, substituting all initial segments (root stems) with connecting bifurcations. The assembly of individual trees into a joined arbor depended on the relative distances of their initial segments of each, with the nearest pair becoming siblings of a new bifurcation located mid-way between them, and the process repeated until only bifurcations remained. Although the same method can similarly handle multifurcations (i.e. 3 or more child branches), NeuroMorpho.Org preprocessing eliminates such cases by splitting them into multiple bifurcations at successive segments. Based on proximity of bifurcations, up to 1.5% of dendrites, 2.8% of axons, and 0.6% of apical dendrite branching events were trifurcations. A minority of datasets included reconstructions of spines labeled as short dendritic branches. To avoid confusing arbor topology with representation of spines, dendrites were further processed by removing any terminal dendritic branch shorter than 2 microns (969 dendritic arbors affected; branches removed: mean of 7.1, median of 2, maximum of 546). This preprocessing step had little impact on the conclusions of this study, but had a notable effect on analysis restricted to larger dendrites, as explained in the Results.

Given this work’s focus on topological pattern detection and analysis, a substantial number of neurites were discarded due to having too few bifurcations and therefore insufficient complexity. Trees of a given size have a limited number of shapes (Figure 3a), that number increasing approximately exponentially with tree size (Figure 3b). We therefore picked a minimum tree size in order to maximize the available data while minimizing the chance of two unrelated trees having identical topology. At a size of 20 bifurcations, the odds of two trees in the dataset matching by chance (N = 276) is about 5%; the odds halve at a size of 21 (N = 280), with the chances still less than 25% if allowing for a single edit (insertion or deletion of a branch) from a tree of size 21. Using a minimum tree size of 20 bifurcations (inclusive), 1,056 axons, 2,460 dendrites, and 1,588 pyramidal apical dendrites were excluded. The final dataset contained 6,798 neurites in total, with 1,255 axons, 4,686 dendrites, and 857 pyramidal apical dendrites.

**Figure 4:**
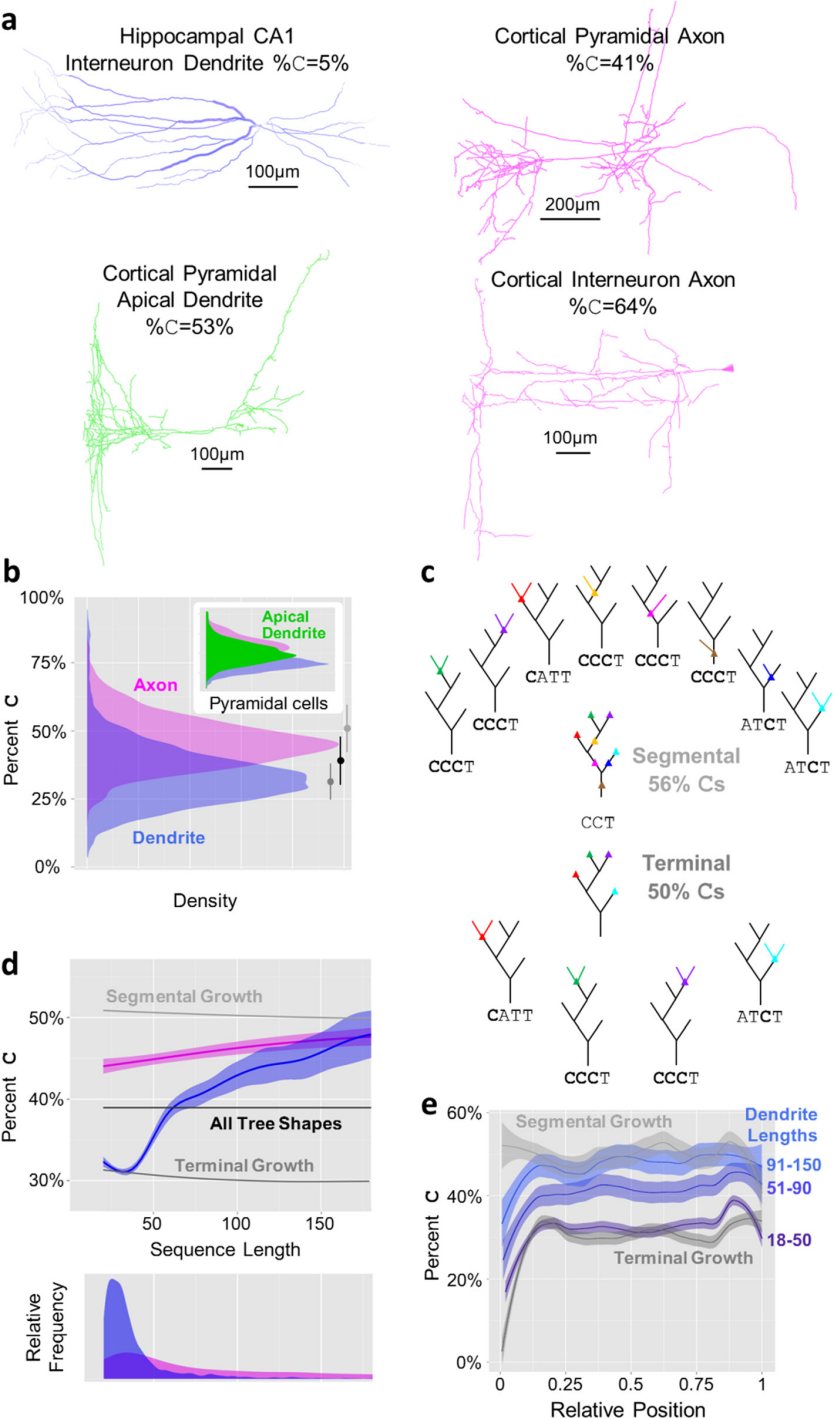
Neurite size and node type features. **a**. Example morphologies along with node-type proportions (%*C*) illustrate how difficult it is to estimate topological patterns by visual inspection of full morphologies [65-67]. **b**. Percent *C* distributions of axons (magenta) and dendrites (blue) overlap but are clearly distinct. Inset: Pyramidal cell basal apical dendrites (green) fall between basal dendrites and axons, which respectively are similarly distributed to the (non-apical) dendrites and axons of all (non-pyramidal) neurons. Biased terminal (dark gray) and segmental (light gray) growth bound the neurite populations, with the unbiased distribution of tree shapes (black) falling in between. **c**. Schematics of terminal (top) and interstitial (bottom) growth starting from a representative seed tree shape with sequence *CCT*. Colored dots represent potential bifurcation points given the growth mechanism, with their respective resulting branches seen in the trees surrounding the initial tree. Segmentally grown trees contain more *C* bifurcations than terminally grown trees on average, though the percentages stabilize at lower values (seen in panel **b**) at around 15 bifurcations. **d**. Percent *C* versus sequence length for axons and dendrites compared to the tree shapes baseline, segmentally grown trees, and terminally grown trees with low-order bias. Axons fall in between segmental and terminal growth while the bulk of dendrites display terminal growth followed by a possibly segmental growth-based rise in %C with larger sequence lengths. Below: Distribution of sequence lengths for axons and dendrites. **e**. Percent *C* as a function of relative position within sequences for dendrites of several sequence size groups along with segmental and terminal growth. The increase in %*C* with sequence length is sequence-wide and not specific to distal portions of trees. The initially low %*C* and rise to stability is similar to that displayed by terminally grown trees.

The content of NeuroMorpho.Org is representative of the species, brain regions, and cell types that are commonly studied and reconstructed [36]. After the above described selection, in the dataset used here Human data made up 37% of dendrites, Mouse 28%, and Rat 23%. Axons were made up of 50% Rat, 35% Mouse, and 11% *Drosophila*. Brain regions were represented primarily by Neocortex in both axons and dendrites, followed by Hippocampus and *Drosophila* Olfactory bulb in axons, and Hippocampus and Retina in dendrites. Apical dendrites came primarily from Rat (53%), Mouse (35%), and Monkey (10%), with 68% from the Neocortex and 31% from Hippocampus with nearly equal proportions between CA1 and CA3. Although the data is heterogeneous and further motif distinctions can be made between species and cell types of a given arbor type, this dataset reliably captures general research trends in terms of arbor type and metadata distribution, and thus serves effectively as a test bed for topological sequence analysis.

## Results

### Length and node-type proportions across arbors types

Many morphological features of neurons, particularly the arbor spatial coverage, total wiring length, and typical branch diameter, can be visualized and roughly estimated simply by looking at a reconstruction. Some topological features involving similarly straightforward calculations, such as the proportion of node types, are often quite difficult to discern visually (Figure 4a). Since the number of *T* nodes (*#T*) is always one more than the number of A nodes (*#A*), node type proportion information is reported compactly as the sequence length and proportion of *C* nodes (%*C*).

$$ \begin{array}{l} Length=\#A+\#C+\#T\\ {}\#T=\#A+1\\ {}\#A= Length-\#C-\left(\#A+1\right)=\frac{Length-\#C-1}{2}\\ {}\%A=\frac{A}{Length}=\frac{1-\%C\raisebox{1ex}{$1$}\!\left/ \!\raisebox{-1ex}{$ Length$}\right.}{2}\\ {}\%T=\frac{1-\%C+\raisebox{1ex}{$1$}\!\left/ \!\raisebox{-1ex}{$ Length$}\right.}{2}\end{array} $$

Axons and dendrites show significantly different distributions of bifurcation type proportions, measured by the percentage of *C* nodes (%*C*), reflecting different functional properties and growth mechanisms (Figure 4b). Pyramidal cell axon and dendrite distributions are broadly similar to the overall trends, but show a broader axonal distribution. Apical dendrites have %*C* values that fall between axons and (basal) dendrites. These distributions are highly correlated with asymmetry (R^2^ of 0.91 for dendrites, 0.89 for axons, and 0.85 for apical dendrites); however, apical dendrites have an asymmetry distribution that almost completely overlaps axons while their %*C* distribution falls below axons and above dendrites (Additional file 1: Figure S1). Meanwhile, their caulescence distribution falls above both axons and dendrites, similar to maximum branch order. This indicates that the topological complexity captured in motifs is not fully captured by traditional metrics.

The lower %*C* values of (non-apical) dendrites and the high values of the axons are suggestive of two conceptual growth models focusing on the site of bifurcation, namely terminal growth and segmental growth [21]. Terminal growth consists of a model neurite bifurcating only at terminal segments, reflecting growth cone bifurcation, for some predetermined tree size (see schematic in Figure 4c). A segmental growth model allows bifurcations to occur with equal probability at terminal and interstitial branches. The %*C* distribution of dendrites matches fairly closely to the values of trees generated by a terminal growth model. Specifically, a small bias towards bifurcating at low-order branches (see Methods) is sufficient to achieve the precise overlap of low %*C* (Figure 4b,d). Conversely, axons more closely fit the segmental growth model.

Most dendritic arbors have relatively few branches (Figure 4d - bottom), but for those that are larger than 40 bifurcations, the %*C* trends upwards until matching the %*C* distribution of axons at approximately 150 bifurcations (Figure 4d). This trend was systematic across all cell types consisting of a sufficient number of large dendrites (without removing likely spines from reconstructions, the trend had an even steeper slope and rose well beyond %*C* of axons). We initially speculated that this effect could be due to an increase in *C* bifurcations farther out in dendrites; however this hypothesis is proven false by analyzing the %*C* as a function of sequence position (Figure 4e). The only position effect, also observed in trees generated by terminal growth, is seen at the beginning and does not change substantially in larger dendrites. This suggests that larger dendrites are primarily larger by virtue of a segmental growth process and a greater proportion of *C* bifurcations.

Axons show only a very small change in %*C* with sequence length. This change could be an artifact of incomplete reconstructions. Axons generally cover far greater areas relative to dendrites and thus are commonly only partially reconstructed. The relative stability of the %*C* suggests that branching patterns might be largely unaffected by partial reconstruction; however, as discussed below, dimer and trimer trends in axons do show a tree size effect.

### Motifs and anti-motifs

**Figure 5:**
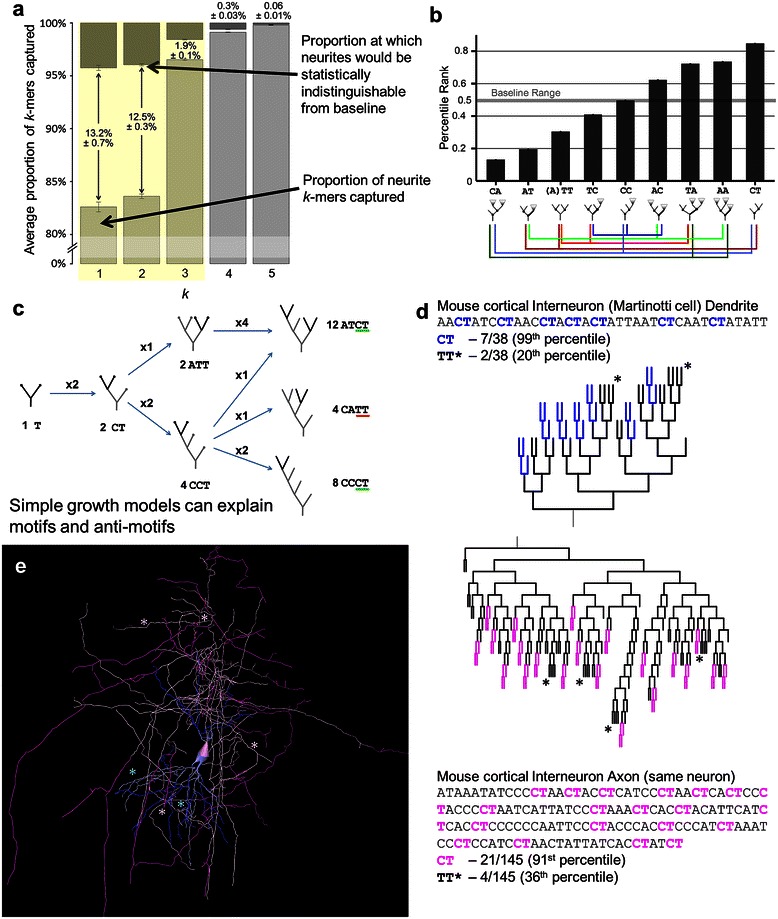
Dimer analysis reflects terminal growth effects. **a**. Average proportions of captured *k*-mers (percentile rank between 2.5^th^ and 97.5^th^) at each *k* for all neurites, with a break between 0 and 80%. Over 95% of trimers are captured, as are over 98% of tetramers, suggesting that most analyses should focus up to dimers, and possibly trimers. Darker descending bars represent the baseline, or chance level based on bootstrapping of surrogates. Numbers between or above bars signify the gap in percent captured between real neurites and the baseline (i.e. statistical equivalence). **b**. All axon and dendrite dimers except *CC* are motifs or anti-motifs. Colored lines below dimer schematics show associations between dimers. When one dimer proportion increases (and in turn its percentile rank) another must decrease. **c**. Growth processes produce specific topological patterns. Starting with a tree of a single bifurcation, the tree grows one bifurcation at a time from terminal nodes. After two bifurcation steps the *CT* to *TT* ratio is 2:1 as there are twice as many ways a *CCT* shape can emerge compared to an *ATT* shape. After a third bifurcation (at right), the dimer ratio is 5:1, and still 3:1 controlling for %*C*. **d** and **e**. *CT* and *TT* motifs and anti-motifs shown in an exemplar interneuron’s dendritic and axonal arbors (NMO_00340 from [68]) in sequence and dendrogram **(d)**, and full morphology **(e)**. Sequence and dendrogram highlighting indicate *CT* dimers in the axon (pink) and dendrite (blue). In the morphology, the darker color indicates the *CT* dimers. Asterisks (*) indicate the *TT* dimer in both representations. *All error bars are standard error of the mean.*

When analyzing *k*-mers of increasing length, it is expected that at some *k* there will be little if any additional information beyond the patterns already identified with shorter sub-sequences. In order to determine at what value of *k* the analysis should conclude, we identified the proportion of *k*-mers that were predicted by the *(k-1)*mer-constrained baseline for each *k*. If the PR fell within the middle 95% (2.5% < PR < 97.5%) then it was considered “captured” by the baseline. Trimer proportions almost fully constrained the representation of neurite tree sequences such that 99.1% of tetramers were captured (Figure 5a). As the capture rate of an additional set of trimer-constrained surrogate tetramers (distinct from the set used for normalization) was 99.4%, the gap between real neurite tetramers and the additional surrogates was 0.3 ± 0.03% (SEM). The additional *k*-mer constrained surrogate set was necessary due to the discrete nature of the *k*-mer distributions. Note that rather than a surrogate set achieving 95% of *k*-mers captured as would be expected with continuous data, the capture rate increases with *k* because more baseline count distributions have either 0 variance or the top or bottom of the distribution is composed of only a single value. The results are similar across arbor types at *k* = 4, with axons showing the lowest capture rate and largest gap between neurites and baseline of 98.1 ± 0.6% and 0.6 ± 0.1%, respectively. Although the difference between neurite and baseline was statistically significant due to the large *n* of over 6,000 sequences, the negligible effect serves to signal a cutoff for analysis beyond trimers. In other words, describing the neurite distributions of monomers (%*C*), dimers, and trimers is also sufficiently informative of the distribution of longer patterns (capturing e.g. all but 0.3% of tetramers).

Except for one (*CC*), each of the 9 dimers is either a motif or an anti-motif, primarily reflecting terminal growth (Figure 5b), in contrast to the node type results (monomer pattern) observed for axons. Percentile rank values differ by a significant but small amount between axons and dendrites, with pyramidal apical dendrites deviating from baseline in the same direction but by far smaller amounts for each *k*-mer (Additional file 1: Figure S2). Because *k*-mers for a given *k* are not independent of one another, a more detailed analysis requires a grouped approach. A *k-*mer that diverges from baseline substantially more than its counterbalanced *k*-mers, such as *CA* compared to *AA* and *TA*, is particularly illuminating. In this case, *A* nodes tend to descend from other *A* nodes, either on the small (*AA*) or large side (*TA*), and are particularly unlikely to be children of *C* nodes. Thus, axons and (non-apical) dendrites have highly arborizing regions topologically close to the soma, while the *CT* motif indicates that *C* nodes tend to occur primarily near neurite subtree terminations. In comparison, apical dendrites have much less extreme values for all dimers, better reflecting a segmental growth mechanism and a more even distribution of *C* nodes throughout the tree.

The strong *CT* motif and strong *TT* and *AT* anti-motifs are at least in part due to the greater number of growth pathways that produce trees with *CCT* or *ATCT* compared to *ATT* subtrees (Figure 5c). Increasingly complex terminal growth examples show even larger proportions of *CT* dimers relative to *AT*, while segmental growth shows a balance of the two while still maintaining lower than baseline proportions of *TT* dimers. The *CT/TT* effect is consistently observed in dendrites and axons from a variety of neuron types, and particularly Martinotti interneurons (Figure 5d-e).

Trimer patterns generally continue several trends revealed by dimers including the nearer-to-baseline profile of apical dendrites and the similarity between dendrites and axons. Of the 39 trimers, dendrites and axons share 9 anti-motifs and 11 motifs (Additional file 1: Figure S3). Trimer motifs *CAT* and *CCT*, and anti-motifs *AAT* and *CCA*, are consistent with primarily terminal growth and trees with *C* nodes associated with terminal *T* nodes. The *AT* dimer, a single branching event from a *C* node, is also seen as associating with *C* nodes in motifs *CAT* and *ATC* rather than with arborizing A nodes as evidenced by anti-motifs *AAT* and *ATA*.

**Figure 6:**
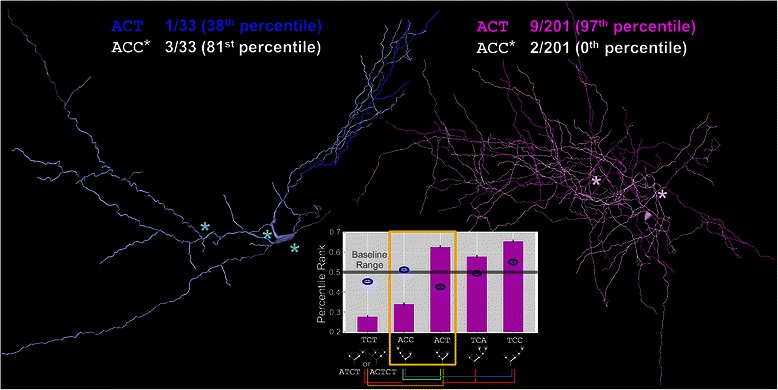
Distinguishing trimers of axons and dendrites. Axons and dendrites share some motifs consistent with dimers and their interpretations, but they differ on a small related set of *k*-mers. A rat hippocampal CA3 interneuron dendrite (left*;* NMO_00837 from [69]) displays a relative abundance of *ACC* trimers (asterisks) but only one *ACT* occurrence (dark blue). A rat cortical basket cell axon (right; NMO_07461 from [70]) has 9 occurrences of *ACT* (magenta) but only 2 of *ACC* (asterisks). Graph Inset: The *ACT* *k*-mer is a motif for axons (magenta bars) and a slight anti-motif for dendrites (blue ovals), while *ACC* is an anti-motif for axons and neither motif nor anti-motif for dendrites.

A particularly distinguishing feature that sets axons and dendrites apart manifests in a small collection of trimers (*TCT*, *ACC*, *ACT*, *TCA*, and *TCC*) that show a tendency in axons for larger-side subtrees to continue rather than terminate (Figure 6). Specifically, while both axons and dendrites have *CCT* as a motif, only axons have *TCT* as strong anti-motif and *ACT* as a strong motif. *TCT* represents complete subtrees *ATCT* or *ACTCT* (since the latter subtree must be of equal or larger size) and is counterbalanced by *TCA* and *TCC* which allow for larger-side subtrees of increased size. The *ACC* anti-motif is most simply explained as a counterweight for the *AC* *T* and *T* *CC* motifs. This difference between axons and dendrites is at least in part due to their different tree sizes, as these trimers are among several that become more extreme with sequence length (consistent with terminal growth effects).

The effect of sequence length raises the question as to whether other trimers might in fact differ between axons and dendrites. Indeed, multiple dimer and trimer PRs change with sequence length in both axons and dendrites, as well as in virtual trees generated via terminal and segmental growth. When controlling for this effect, axons partially diverge from dendrite dimer and trimer motifs, coming more into agreement with the %*C* profile intermediate between terminal and segmental growth. For instance, segmentally grown trees’ *CAT* PRs fall near the baseline (50%), while in terminally grown trees, in axons, and in dendrites they start near the baseline and then rise considerably (Additional file 1: Figure S4). However, across sequence lengths (until the shift in dendrite topology at about 50 bifurcations) axons maintain a lower normalized proportion of *CAT*. Their larger average sequence length compared to dendrites then causes the average PRs of the arbor types to be so similar. The changing topology of growing trees thus requires consideration of proportions by sequence length for a complete analysis.

While we chose to analyze all unique *k*-mers from *StL* and *LtS* traversals, it could also be reasonable to exclude “composite” *k*-mers consisting of multiple subtrees not connected within the *k*-mer. Such *k*-mers include 10 trimers and 45 tetramers (for instance *CTC*, *ATTA*), but not those such as *ATC* for which the *C* is connected to the *A*. Composite *k*-mers would be difficult to interpret, and they do not correspond to any connected structure within a tree. When we excluded them both as constraints and in the percentile rank analysis, the results changed negligibly. The percent of trimers not captured by dimer constrained surrogates increases from 1.9% to 2.9% ± 0.1% (SEM). For tetramers the capture rate rose from 0.30% to 0.32% ± 0.03%, and for pentamers the rate decreased from 0.066% to 0.056% ± 0.01%. These minimal alterations show that our conclusions are unaffected by the inclusion of composite *k*-mers.

## Discussion

The novel method of representing neuronal trees as sequences of bifurcations introduced in this work facilitates detection and analysis of branching patterns. Using an exclusively topological approach we show that sequence length and subsequences up to three characters long almost completely capture the local topology of neuronal axons, dendrites, and apical dendrites.

Node type distributions highlight the most substantial differences between axons and dendrites, with axons having higher proportions of terminal side-branches (*C* nodes). The vast majority of dendrites exhibit particularly low proportions of *C* nodes, but the proportion increases considerably for dendrites with greater than 40 bifurcations. Dimer and trimer motifs show much greater similarity than difference between axons and dendrites, highlighting a primarily terminal growth effect with some particular and likely complex segmental growth process occurring in axons and larger dendrites. Pyramidal neuron apical dendrites (mainly cortical), on the other hand, stand out as having a *k*-mer profile very similar to a basic segmental growth model with relatively small deviation from the baseline of tree shapes.

### Biological interpretations

Dendrites have generally denser spanning fields than axons and the functional role in processing inputs from other neurons can further constrain their shape. While homogenous targeting can lead to winding and asymmetric trees in models that only minimize neurite wiring, efficient signal integration (i.e. current transfer to the soma) demands shorter paths, which in turn require more symmetric trees [9]. It is tempting to hypothesize that terminal growth processes are simpler or more efficient at producing dendrites satisfying such constraints, and thus the dendritic motif profile reflects those dominant features. Pyramidal apical dendrites, particularly those in the neocortex, have a more heterogeneous spanning field, with relatively sparse coverage along the trunk and denser coverage in the distal tuft [37,38]. Segmental growth may better fit such a target region shape, though biased retraction following initial terminal growth, which is known to take place for some differentially projecting pyramidal subtypes [39], is an additional factor that impacts topology [40]. The molecular and biophysical specialization of the apical dendrite [35,41] optimizes input integration by minimizing the effective path distance of farther synapses [42].

Axon motifs indicate a more complex growth process with clear influence of terminal growth but also with indications of segmental growth. While axons are not as constrained as dendrites in terms of path distance and target region size, they are the neuronal component most responsible for navigating through the environment to create connections. Axons are also characterized by a more substantial energy footprint and specific energy regulation mechanisms [43]. Developmental studies have shown that axons branch interstitially [44]; however, during the interstitial branch outgrowth the growth cones stop [45,46]. In conjunction with axonal self-avoidance [47,48] and retraction during development [49], this complex orchestration might produce terminal growth-like features.

Axon morphology is also impacted by post-developmental activity and plasticity. Axons are motile and plastic, displaying branch growth and retraction in adult animals [50,51]. While this is also true of dendrites, on average axons have much greater inter-branch distance along which to bifurcate or move in response to environmental cues. These capabilities have important implications for network plasticity and learning, including the possibility of branch outgrowth in response to local network activity. Such branching would be segmental in nature since it could occur at any location along an axon; such a mechanism would at least partially explain the high %*C* in axons, which otherwise display topological patterns largely indicative of terminal growth.

The motif results of axons will benefit from confirmation with a larger set of complete reconstructions, as many axons in the dataset are likely incomplete [52]. However, the relationship between k-mer percentile ranks and sequence length generally followed the trends seen in a basic terminal growth model. Assuming axon tree size as a proxy for completeness, it is likely that the general findings will be confirmed. Nonetheless, subsets of the axon data may reflect reconstruction artifacts.

### Data biases

Given that our analyses primarily focus on arbor types across cell types and brain regions, the results are susceptible to bias in the dataset. Our exploration of potential distinctions between cell types in dendrites revealed few clear differences beyond sequence length distributions given the sample sizes available. The same appears to hold for axons, though the sample size of diversity of neuron types is far more limited. Analysis in the companion study do show substantial global differences between four axon subtypes, with only modest motif profile differences between three of them [53]. This suggests that the results are generally applicable for dendrites and axons, and that most differences between classes, while potentially interesting and worth further study, are small compared to deviations of the arbor types from the baseline.

Apical dendrites, on the other hand, show substantial differences between brain regions. The results presented here are dominated by the more numerous neocortical pyramidal cells. The hippocampal CA1 and CA3 motif profiles differ from the neocortical profile and from each other, consistent with known morphological and anatomical differences between the three regions.

### Baselines

The choice of baseline necessarily impacts whether a *k*-mer is a motif, an anti-motif, or neither. We chose to use the set of tree shapes, sampled uniformly, as a baseline in order to minimize any assumptions regarding neurite topology. While our analysis focused on absolute motifs using the single baseline, differences between groups can be analyzed given the comparable nature of the *k*-mer percentile ranks.

Future analyses which focus on deviations from a given growth model might benefit from using that growth model as a baseline. For instance, further elucidating axonal growth programs might use a terminal growth model baseline. Such an approach would still require analysis across sequence lengths as different growth processes can produce different changes in topology with tree size. Moreover, normalizing *k*-mer proportions by a given growth model would require new algorithms for generating the surrogates with *k*-mer constraints. Simply growing the trees and then only accepting those that satisfy the constraints is not practical for large trees, which most axons are. Alternatively, a set of terminally grown trees could have its own set of surrogates and normalized proportions (i.e. percentile ranks; Additional file 1: Figure S4), and the neurite dataset motifs could be defined by their deviation from the terminal growth normalized proportions.

### Alternative encodings and traversals

Although the current work focused on purely topological encoding, the approach to represent arbors as strings of characters can be extended with additional geometric characteristics of branches. Several branch-level features might be discretized and encoded, such as branch length, tortuosity, or bifurcation angle. With an expanded sequence based on a larger alphabet of characters, the number of *k*-mers would increase dramatically. Motif analysis could allow us to determine whether certain branch-level features co-occur with each other and with specific topological patterns, providing novel observations for forming new growth and functional hypotheses.

Classification is another potential use of any new representation and measure. In the case of topological motifs the variability is too large to be particularly useful in classification; however, other analyses using this representation as well as an expanded encoding have the potential to aid in classification. A classification example and candidate encodings are discussed further in the companion paper [53].

Rather than a depth-first traversal, a tree could alternatively be encoded as a collection of sequences, each representing a path from root to tip [54]. The utility of the representation likely depends upon the specific investigative aim. One potential draw-back is that low-order branches will be represented multiple times, which, in addition to imposing a bias for certain types of analysis, multiplies the size of the representation. A benefit of such a representation lies in preserving the sequential pattern of all paths.

### Model validation and additional applications

The current topological representation, along with expanded representations, could prove valuable as a more sensitive measure of emergent topology relative to standard morphometrics for gauging the quality of computational simulations of developmental processes. Standard topological metrics are useful for determining whether a particular model matches the true distribution, but motifs may provide a clearer indication of how the model and true neurite distributions diverge and what modifications might be necessary to improve the model. Experiments focusing on functional constraints, such as wiring efficiency or distribution of synaptic targets, would have the added benefit of more clearly associating motifs with neuronal function. Though not detailed here, the distinctive motifs of neurites from different neuron types suggest that topological arbor patterns may be impacted by the specific functions and anatomic contexts of neuron types.

### Alternative motif definitions

While our analysis involves motifs of the variety commonly discussed in graph and network analysis [32], the definition of motifs used here is one of several used in computational biology. Network analyses commonly seek out combinations of interactions between network elements that occur more frequently than in a random or other baseline network. Such motifs can indicate the type of network based on their mechanism of generation and/or functional characteristics. In genomics, sequence motifs refer to short, highly conserved segments of nucleotides often functioning as binding sites [55]. Structural protein motifs are commonly occurring combinations of secondary structure elements (e.g. beta hairpin or helix-turn-helix) [56].

More broadly, motifs can be defined as any commonly occurring pattern within an appropriately defined set of structures. BlastNeuron [57] uses topological and spatial alignments of neuronal morphologies to detect morphological clusters, and defines motifs as the major branches shared by most or all neurons in a cluster. These motifs are akin to the consensus sequences in the companion study generated via multiple alignment of clustered topology-encoded neurite sequences [53].

## Conclusions

The proposed sequence representation of binary trees is effective for quantifying the topological patterns of neuronal arbors. Motif analysis offers a measure to validate or refine models and hypotheses. It reveals that the local topological features of both axons and non-apical dendrites are similarly consistent with terminal growth-like processes despite substantially different overall size and function. In contrast, pyramidal apical dendrites exhibit a motif profile indicative of segmental growth.

The methods described here and in the following paper can generally be applied to any tree structure of sufficient complexity. Research in glia [58,59], brain vasculature [60], botany [61], rivers/watersheds, and phylogenetics are all potential targets of a sequence analysis approach. Moreover, while the topological sequence representation is powerful as it is, it also can serve as a basis on which other morphological features are added.

## Additional file

Additional file 1:
**Four supplementary figures referenced in the main text.**
