# Supplementary material for: Topological characterization of neuronal arbor morphology via sequence representation: I - motif analysis
Source: BMC Bioinformatics. 2015 Jul 10;16:216. doi: 10.1186/s12859-015-0604-2 (PMC4496917; doi:10.1186/s12859-015-0604-2)
Supplement: Additional file 1: — Four supplementary figures referenced in the main text. [file 12859_2015_604_MOESM1_ESM.pdf]

Supplementary Figures

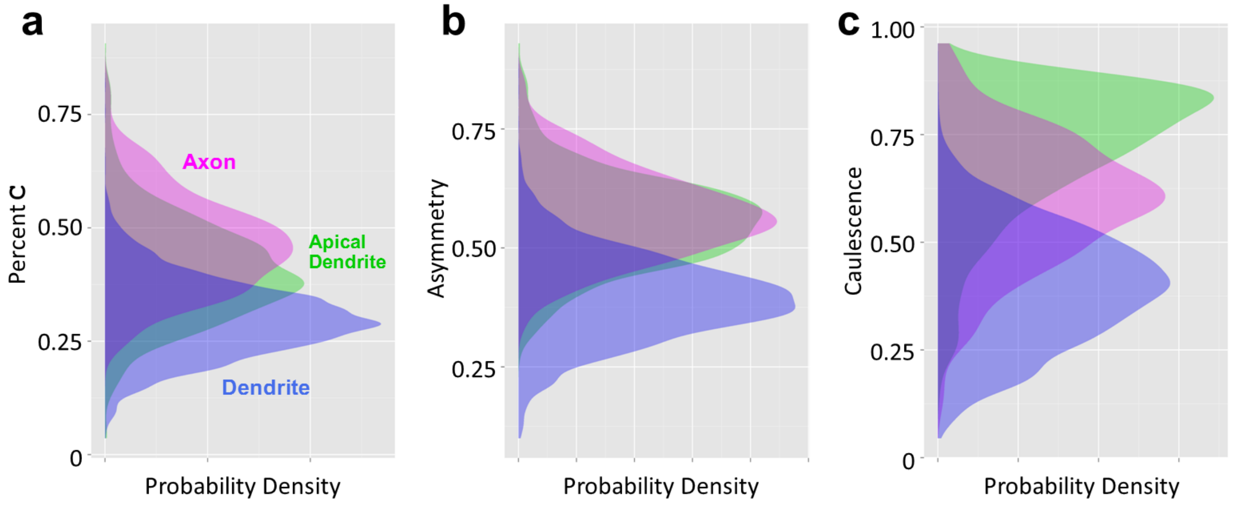

Supplementary Figure 1: Percent C, Asymmetry, and Caulescence. Distributions of %C (**a**), average partition asymmetry (**b**), and caulescence (**c**) for pyramidal axons (magenta), dendrites (blue), and apical dendrites (green). The relationship between axons and dendrites is consistent between measures, but the apical distribution is unique in each.

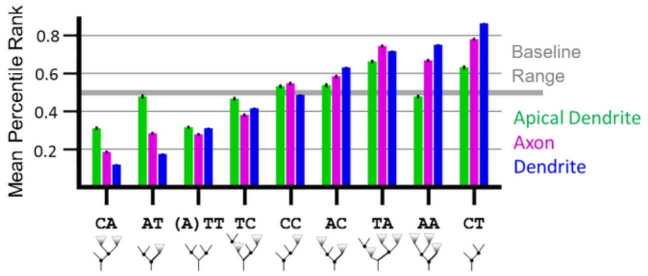

Supplementary Figure 2: Dimer motifs and anti-motifs by arbor type. All mean dimer percentile ranks (with SEM error bars) are shown for each arbor type. Apical dendrites are closer to the baseline (50%) than axons and dendrites except for the case of (A)TT where all arbor types are approximately the same.

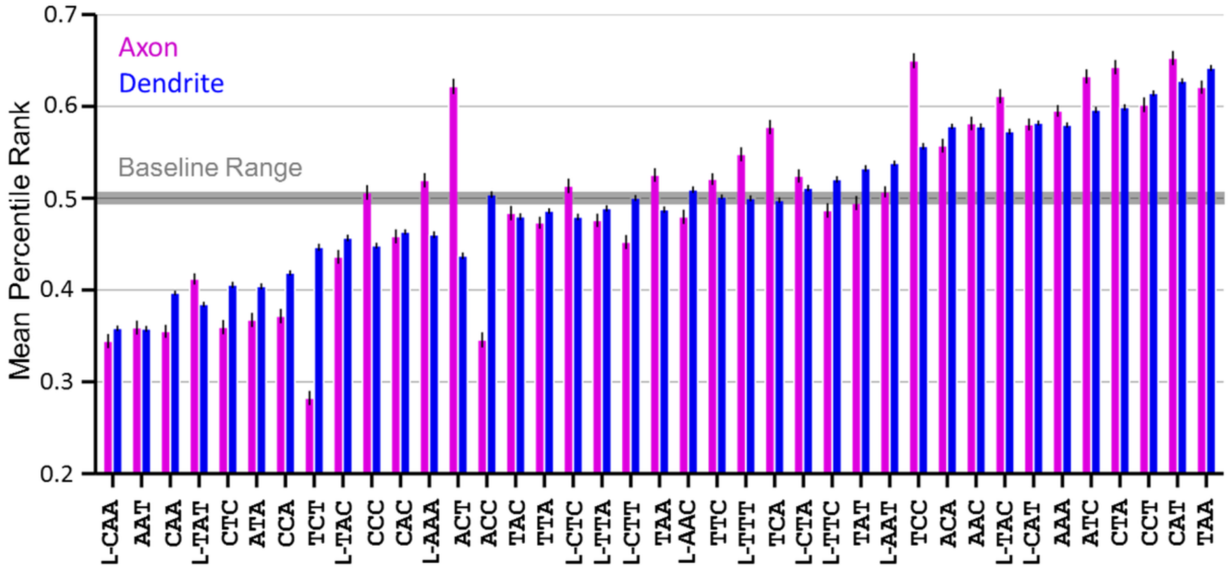

Supplementary Figure 3: Axon and dendrite trimer motifs and anti-motifs. Axons and dendrites exhibit highly similar trimer patterns, with a few notable exceptions. Approximately one-third of trimers are motifs and another one-third anti-motifs.

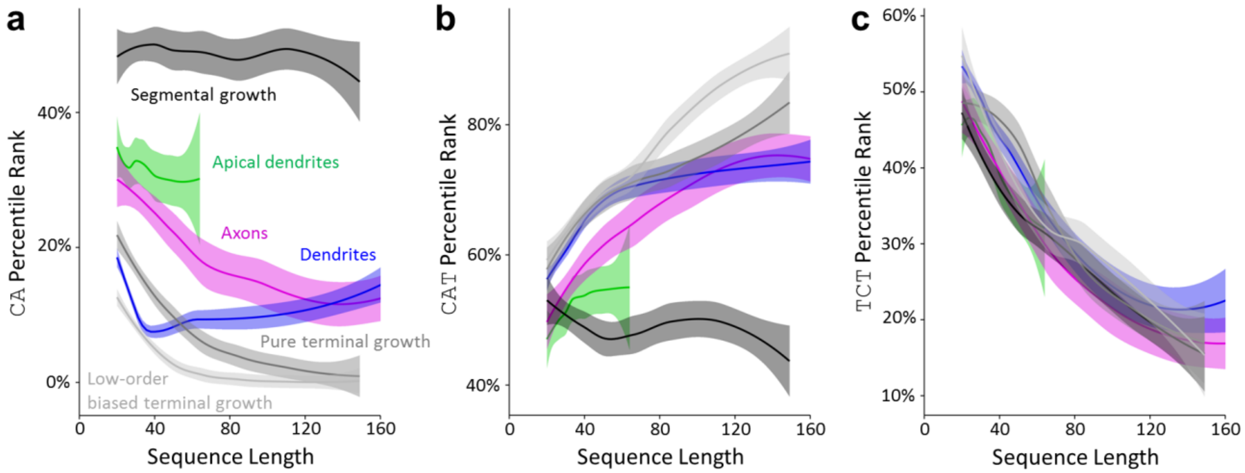

Supplementary Figure 4: Dimers and trimers by sequence length. Percentile ranks of arbor types and growth models (segmental, terminal, and low-order biased terminal), smoothed across sequence length, with 95% confidence intervals, for *k*-mers (**a**) `CA`, (**b**) `CAT`, and (**c**) `TCT`. In the `CA` and `CAT` cases, the different percentile rank trends and different average lengths of axons and dendrites cause the averages across sequence lengths to appear more similar. `TCT`, on the other hand, shows substantially greater similarity of the arbor types and growth models by sequence length, but greater differences when averaged across sequence lengths.
